# Surface Plasmon Resonances in Silver Nanostars

**DOI:** 10.3390/s18113821

**Published:** 2018-11-08

**Authors:** Faustino Reyes Gómez, Rafael J. G. Rubira, Sabrina A. Camacho, Cibely S. Martin, Robson R. da Silva, Carlos J. L. Constantino, Priscila Alessio, Osvaldo N. Oliveira, J. Ricardo Mejía-Salazar

**Affiliations:** 1 Departamento de Física, Universidad del Valle, AA 25360 Cali, Colombia; faustino.reyes.gomez@gmail.com; 2 Department of Applied Physics, University of Cantabria, Avda. Los Castros, s/n, 39005 Santander, Spain; 3 School of Technology and Applied Sciences, São Paulo State University (UNESP), Campus Presidente Prudente, São Carlos 19060-900, Brazil; rafael.gon.fis@gmail.com (R.J.G.R.); sabrina.alessio@gmail.com (S.A.C.); cssmartin@gmail.com (C.S.M.); carlos.constantino@unesp.br (C.J.L.C.); priscila.alessio@unesp.br (P.A.); 4 São Carlos Institute of Physics, University of São Paulo, PO Box 369, São Carlos 13560-970, Brazil; robsilva31@iq.unesp.br (R.R.d.S.); chu@ifsc.usp.br (O.N.O.J.); 5 National Institute of Telecommunications (Inatel), Santa Rita do Sapucaí, MG 37540-000, Brazil

**Keywords:** metallic nanoparticles, plasmonic biosensing, Ag nanostars

## Abstract

The recent development of silver nanostars (Ag-NSs) is promising for improved surface-enhanced sensing and spectroscopy, which may be further exploited if the mechanisms behind the excitation of localized surface plasmon resonances (LSPRs) are identified. Here, we show that LSPRs in Ag-NSs can be obtained with finite-difference time-domain (FDTD) calculations by considering the nanostars as combination of crossed nanorods (Ag-NRs). In particular, we demonstrate that an apparent tail at large wavelengths (λ≳700 nm) observed in the extinction spectra of Ag-NSs is due to a strong dipolar plasmon resonance, with no need to invoke heterogeneity (different number of arms) effects as is normally done in the literature. Our description also indicates a way to tune the strongest LSPR at desired wavelengths, which is useful for sensing applications.

## 1. Introduction

Metallic nanoparticles (NPs) of different sizes and shapes have found applications in many fields such as in optics, photovoltaics, biological imaging, cancer therapies, for the environment, among others [1,2,3,4,5,6]. This interest has been mostly due to the near-field enhancement on the particles surface through excitation of localized surface plasmon resonances (LSPRs), which has been exploited in ultrasensitive biosensing with surface-enhanced Raman spectroscopy (SERS) [7,8,9,10,11,12,13,14,15,16,17,18,19,20,21] and surface-enhanced infrared absorption (SEIRA) spectroscopy [22]. In SERS, chemical and biological analytes can be probed down to the single molecule level [23], with enhancement factors (EFs) depending on the size, shape, and chemical composition of metallic NPs [9,10,11,12,15,19]. This dependence has motivated the synthesis of metallic NPs with different morphologies [13,24], especially the highly anisotropic NPs such as nanostars (NSs) and nanoplates (NPLs), where the field enhancement of the tip plasmons may result in more efficient SERS [25]. In one of the latest advances [13], silver nanostars (Ag-NSs) have provided high SERS performance [26,27,28]. The work in this field has been focused on the control of morphology and empirical mechanisms to further enhance the electromagnetic field, while less attention has been given to a theoretical interpretation of some of the observed phenomena. For example, the extinction spectra of Ag-NSs display a tail at large wavelengths (λ≳700 nm) which is attributed to nanoparticle aggregation and scattering by different morphologies of Ag-NSs and tip sharpnesses [13,26,27,28].

In this work, we demonstrate with numerical results from finite-difference time-domain (FDTD) calculations that the observed tail in the extinction spectra may be explained by excitation of a strong dipolar plasmon resonance. Moreover, the LSPRs in Ag-NSs can be qualitatively retrieved by considering the nanostars as made by crossed Ag nanorods (Ag-NRs), which can be tuned by controlling the arm-lengths of the Ag-NSs. A comparison is also made with the extinction spectra of Ag nanospheres (Ag-NSPs) and triangular Ag-NPLs, in order to demonstrate the adequacy of the theoretical approach.

## 2. Results and Discussion

### Spectroscopy of Silver Nanoparticles: Experiments and Simulations

**Figure 1:**
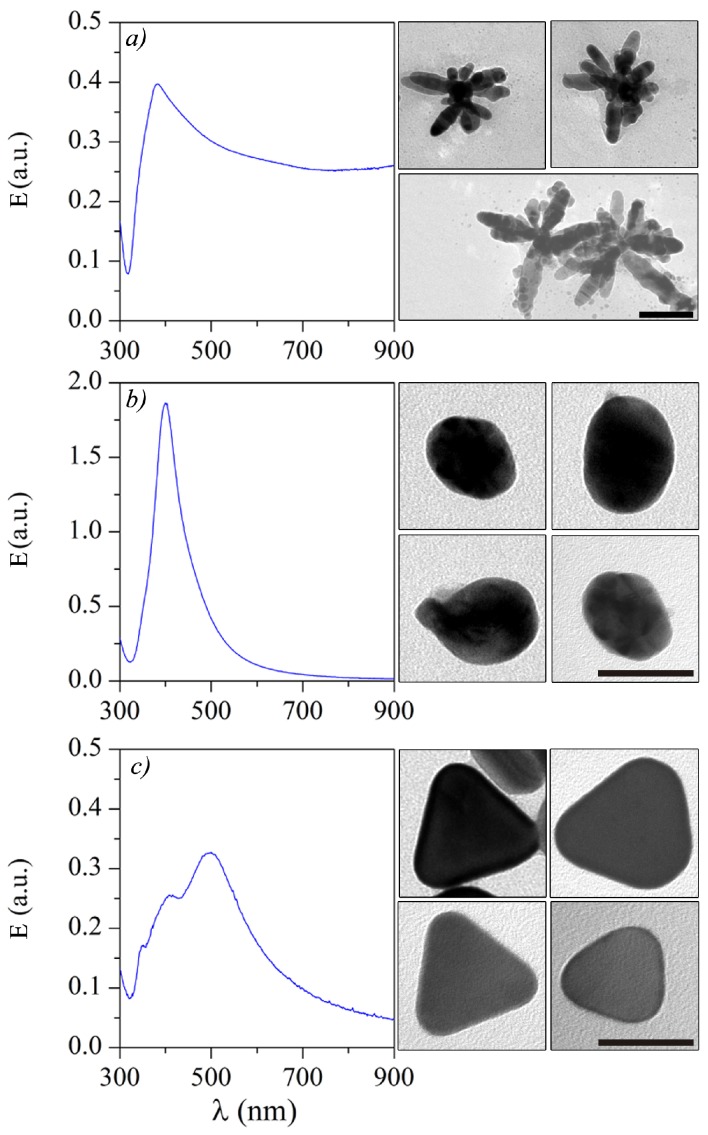
Extinction spectra, *E*, of colloidal suspensions of Ag nanoparticles with different shapes: (**a**) Ag-NSs; (**b**) Ag-NSPs and (**c**) Ag-NPLs, with their corresponding TEM images on the right-hand side. The scale bar is 100 nm for (**a**) and 50 nm for (**b**,**c**).

The UV-Vis extinction spectra, defined as $E=-\log_{10}\left(\frac{I_{T}}{I_{0}}\right)$ ($I_{0}$ and $I_{T}$ are the incident and transmitted light intensities in W/m${}^{2}$) [29], for colloidal suspensions of Ag-NSs, Ag-NSPs, and triangular Ag-NPLs, are shown in Figure 1, together with the TEM images from which the average particle sizes were estimated. These sizes are A=110.5±10.8 nm, D=50±6 nm, and L=38±8 nm for the average arms length (A), diameter (D), and sides length (L), for Ag-NSs, Ag-NSPs, and Ag-NPLs, respectively, consistent with the literature [8,12,13,30,31,32,33,34]. The corresponding LSPRs for Ag-NSs, Ag-NSPs, and Ag-NPLs, were found at $\lambda_{\mathrm{Ag}-\mathrm{NSs}}=380$ nm, $\lambda_{\mathrm{Ag}-\mathrm{NSPs}}=402$ nm, $\lambda_{\mathrm{Ag}-\mathrm{NPLs}}=350$ nm, 407 nm, and 496 nm, respectively.

**Figure 2:**
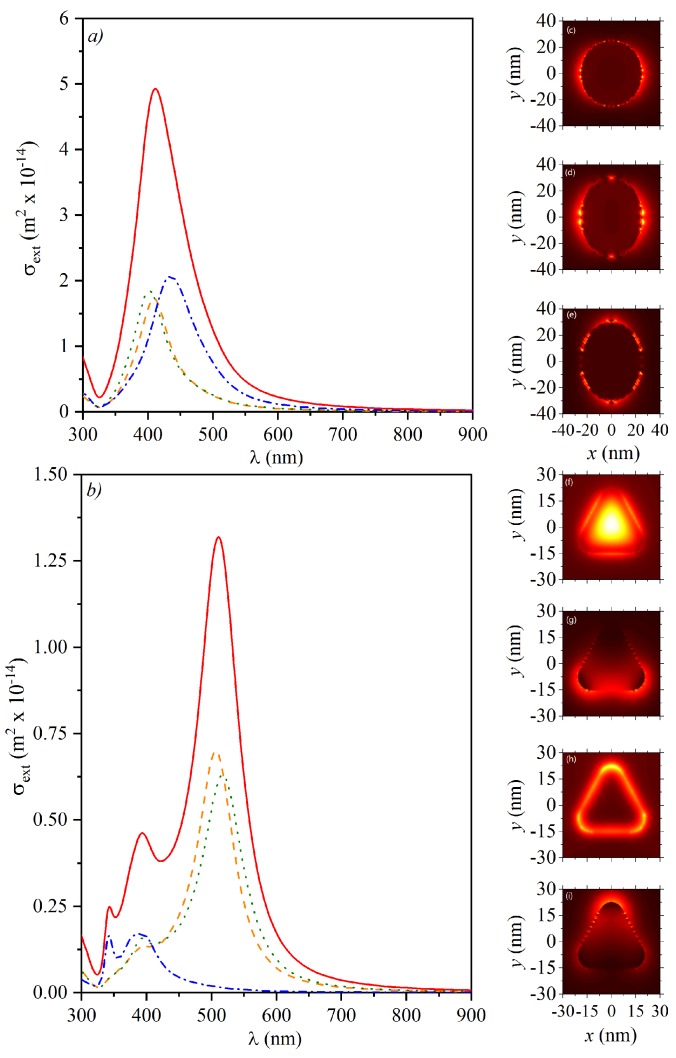
Extinction cross-section spectra, $\sigma_{\mathrm{ext}}$, for (**a**) one Ag-NSP (dashed line), and for an ellipsoid under transversal (dotted line) and longitudinal (dash-dotted line) polarized electric field; and (**b**) for Ag-NPLs under different polarizations along the main axes. Solid lines show the corresponding sum for all the calculated $\sigma_{\mathrm{ext}}$; (**c**–**e**) show the corresponding electric field profiles around the sphere and ellipsoids, as pointed in the insets, at the corresponding resonances; (**f**–**i**) correspond to the electric field profiles for all resonances of the Ag-NPLs. Blue and purple arrows indicate the wavevector and the oscillating electric field, respectively.

**Figure 3:**
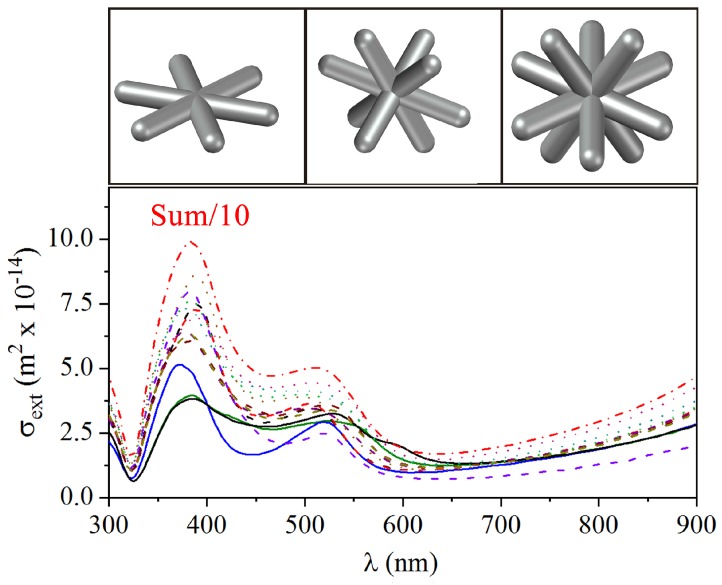
Numerical extinction cross-section spectra, $\sigma_{\mathrm{ext}}$, for Ag-NSs with different numbers of arms and different polarizations of the incident light. The solid, dashed, and dotted curves are for stars with six, eight, and twelve arms, respectively. The dash-dotted line shows the sum of all the calculated $\sigma_{\mathrm{ext}}$ divided by 10 in order to plot within the same scale.

Resonances in the experimental measurements of extinction spectra, *E*, in Figure 1 can be fitted with numerical results for the extinction cross-sections ($\sigma_{\mathrm{ext}}$) in Figure 2 and Figure 3, calculated with the FDTD technique by using the geometrical dimensions from the TEM images. The extinction cross-section is calculated as $\sigma_{ext}=\frac{P_{\mathrm{scat}}+P_{\mathrm{abs}}}{I_{0}}$, where $P_{\mathrm{scat}}$ and $P_{\mathrm{abs}}$ (in units of W) are the total power scattered and absorbed by the particle. Figure 2a shows the calculated extinction spectra for a sphere (with D=50 nm), and for an ellipsoid with semi-minor and semi-major axes a=50 nm and b=56 nm, with circular cross-section under the two main polarizations. Figure 2c–e show the corresponding field profiles, as pointed in the insets in Figure 2a. Figure 2b,f–i show the calculated extinction spectra for a triangular Ag-NPL, with L=38 nm, for field–particle interactions along all the main axes and their corresponding field profiles. The extinction spectra for colloidal suspensions must be a weighted average of the extinction spectra for all the geometries and field–particle interactions in the sample. One should note that the sums of the corresponding extinction spectra are in qualitative agreement with the experimental spectra, since Figure 2a shows the broad peak for Ag-NSPs (resembling Figure 1b) and Figure 2b resembles the LSPR peaks in Figure 1c.

While the spectra for spherical/ellipsoidal and triangular silver nanoparticles have been consistently obtained in the literature with theoretical simulations such as those shown in Figure 2, the same does not apply to the spectrum of the nanostars (Ag-NSs). Indeed, due to the absence of such a theoretical study for Ag-NSs, the tail in extinction at wavelengths λ≳700 nm in Figure 1a has been attributed to a combined effect of agglomeration and different morphologies in the sample [13,27,28]. However, from our simulations in Figure 3, it is clear that both plasmonic bands at around λ=380 nm and λ=529 nm, and the long wavelength tail can be obtained from individual response from stars with six, eight, and twelve-arms, and their combinations, with no need to assume that there is aggregation. In the calculations, we used the arms length as A=110 nm, i.e., the tip to tip length is 220 nm, and a circular cross-section with diameter 38 nm, taken from the TEM images. A tail was present in $\sigma_{\mathrm{ext}}$ for all the Ag-NSs, which clearly indicates that it is not a consequence of a mixture of Ag-NSs with different numbers of arms as supposed in previous works [13,26,27,28]. The more intense band near λ=380 nm in Figure 1a is well captured for all the stars considered and polarizations, but the smaller band in the numerical results is not observable in the experimental results. This latter band does not appear because of the high heterogeneity of morphology of nanoparticles in the sample, which contributes to widening the more intense resonance band. As a consequence, the smaller band is masked. This behavior can be inferred from results in Figure 2 and Figure 3.

**Figure 4:**
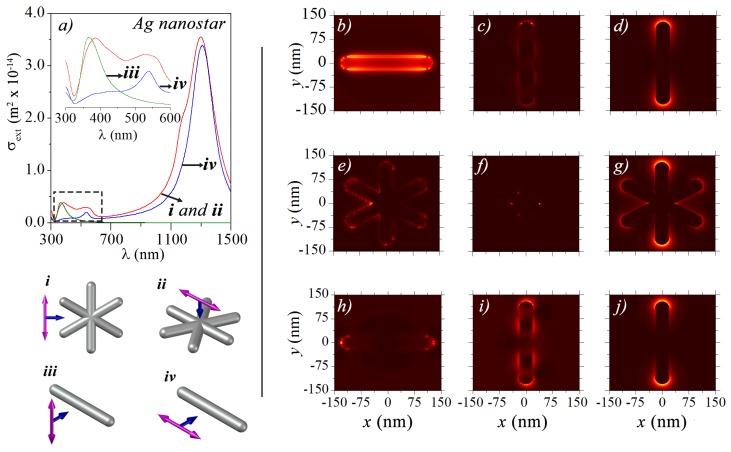
(**a**) red, green, and blue lines correspond to numerical extinction cross-section spectra, $\sigma_{\mathrm{ext}}$, for (*i*)–(*ii*) Ag-NS with six-arms, and for Ag-NRs under (*iii*) transversal and (*iv*) longitudinal electric field polarizations, respectively. Results are presented for Ag-NSs and Ag-NRs with tip-to-tip lengths of 220 nm; (**b**–**g**) show the corresponding electric field profiles around the Ag-NR and Ag-NS at each resonance peak in $\sigma_{\mathrm{ext}}$; (**h**–**j**) show the electric field profiles for the Ag-NS seen from the yz-plane, as a visual aid to compare with the rods.

We show that the apparent tail in the extinction spectra of Ag-NSs at large wavelengths (λ≳700 nm) is due to a residual component of a dipolar plasmon-resonance excitation, which for large tip-to-tip sizes is located outside the UV-Vis region. The red line in Figure 4a corresponds to the numerical $\sigma_{\mathrm{ext}}$ for an Ag-NS with six-arms, with a tip-to-tip length of 220 nm for incident wavelengths up to λ=1500 nm. Excitation of a strong LSPR around λ=1300 nm is observed, owing to the large size of Ag-NSs, which may be relevant for luminescence enhancement of rare earths [35,36] (emitting in the near-infrared), and for theranostic and phototherapy applications [37,38]. For understanding the shape of these resonances, we calculate $\sigma_{\mathrm{ext}}$ for Ag-NRs under transversal and longitudinal electric field polarizations, as shown in the insets in Figure 4, denoted by *iii* and *iv*. The nanorod size was taken as 220 nm according to the tip-to-tip sizes of Ag-NSs. Numerical results for the extinction cross-section spectra of Ag-NRs under transversal and longitudinal polarizations are given in green and blue lines in Figure 4a. Significantly, the spectrum of Ag-NSs can result from the superposition of spectra of Ag-NRs—mimicking the arms—positioned at different directions, as in the inset in Figure 4a that shows a zoom of $\sigma_{\mathrm{ext}}$ for $\lambda \in \left[300\;\mathrm{nm},600\;\mathrm{nm}\right]$. The smaller band for $\sigma_{\mathrm{ext}}$ of Ag-NSs, around 350–380 nm, can be assigned to the quadrupolar plasmon due to the transversal resonance of arms [39], as this mode could not be excited with longitudinal polarization due to symmetry considerations. On the other hand, the octupole-mode around 530 nm can only be excited for sufficiently large Ag-NRs [39], as in the present case. The strongest dipolar plasmon resonance for Ag-NSs, around 1300 nm, resembles the dipolar plasmon resonances for Ag-NRs. Figure 4b–g show the calculated near-field for Ag-NRs and Ag-NSs at their respective LSPRs, while Figure 4h–j show a lateral-side view of the near-field for results in Figure 4e–g. By comparing the near field distributions in Figure 4b–d with the ones in Figure 4h–j, we readily note the similarity in their symmetry properties, as predicted from the results for the extinction spectra.

**Figure 5:**
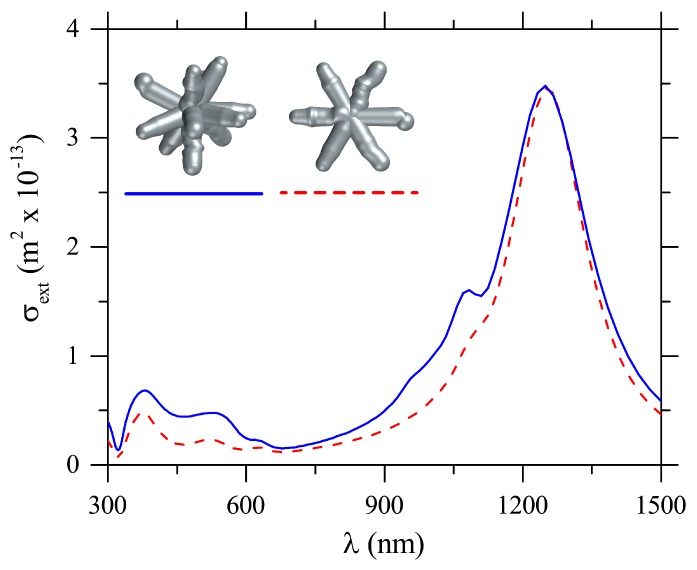
Averaged numerical extinction cross-section spectra, $\sigma_{\mathrm{ext}}=\left(\sigma_{\mathrm{long}}+\sigma_{\mathrm{trans}}\right)$, for rough nanostars with geometries closer to the ones from TEM images in Figure 1a. Arm lengths for both structures range from 110 nm to 140 nm.

A close inspection of Figure 1a points to some roughness of the nanostars, which could in principle modify the extinction spectra. In order to test this hypothesis, we performed additional simulations for rough nanostars whose shapes are closer to the ones observed in the TEM images in Figure 1a. The results in Figure 5 for the averaged extinction spectra $\sigma_{\mathrm{ext}}=\left(\sigma_{\mathrm{long}}+\sigma_{\mathrm{trans}}\right)/2$, however, indicate that roughness effects are negligible.

## 3. Materials and Methods

### 3.1. Synthesis of Silver Nanoparticles

Silver nitrate (AgNO${}_{3}$, MW = 169.88 g/mol), hydroxylamine hydrochloride (NH${}_{2}$OH·HCl, MW = 69.49 g/mol), hydroxylamine solution (NH${}_{2}$OH, MW = 33.03 g/mol, 50% wt/vol), sodium citrate (C${}_{6}$H${}_{5}$Na${}_{3}$O${}_{7}$·2H${}_{2}$O, MW = 294.10 g/mol) and sodium borohydride (NaBH${}_{4}$, MW = 37.83 g/mol) were purchased from Sigma-Aldrich (Cotia, Brazil). Sodium hydroxide (NaOH, MW = 40.00 g/mol) and potassium bromide (KBr, MW = 119.00 g/mol) were acquired from ACP and hydrogen peroxide (H${}_{2}$O${}_{2}$, MW = 34.01 g/mol, 50% wt/vol) from Fisher Scientific (Suwanee, GA, USA). All chemicals were used without further purification. Ultrapure water with resistivity of 18.2 MΩ·cm and pH 5.6, acquired from a Milli-Q Simplicity system (Merck, Darmstadt, Germany), was used in the preparation of colloidal suspensions (silver nanoparticles). The glassware used in the synthesis was cleaned with a sulfochromic solution and rinsed thoroughly with ultrapure water.

The colloidal suspension of Ag-NSs was prepared according to Garcia-Leis et al. [13] via hydroxylamine and sodium citrate reduction. The first step consisted in mixing 500 μL of NH${}_{2}$OH ($6\times 10^{-2}$ mol/L) and 500 μL of NaOH ($5\times 10^{-2}$ mol/L), to which 9 mL of AgNO${}_{3}$ ($1\times 10^{-3}$ mol/L) were added under magnetic stirring for 5 min. The resulting solution became brown. In a second step, 10 μL of sodium citrate (1% wt/vol) were poured into the solution and stirring was kept for 15 min to yield AgNSs colloid with dark gray color. The Ag-NSPs colloid was prepared via hydroxylamine reduction as proposed in Ref. [8], where an aqueous solution containing 4.5 mL of NaOH (0.1 mol/L) and 5 mL of NH${}_{2}$OH·HCl ($43.3\times 10^{-3}$ mol/L) under vigorous magnetic stirring receives 90 mL of AgNO${}_{3}$ ($1.2\times 10^{-3}$ mol/L). The colloidal suspension was obtained after 5 min of continued magnetic stirring. Triangular Ag-NPLs were obtained following the methodologies described by Cathcart et al. [10] and Izquierdo-Lorenzo et al. [12]. In an Erlenmeyer flask, 40 mL of an aqueous solution containing sodium citrate ($2.4\times 10^{-3}$ mol/L), AgNO${}_{3}$ ($1.2\times 10^{-4}$ mol/L), H${}_{2}$O${}_{2}$ ($2.6\times 10^{-2}$ mol/L) and KBr ($6.5\times 10^{-7}$ mol/L) were kept in a cold bath ($∼4{\;}^{\circ }$C) for 30 min without stirring. Then, the flask was removed from the cold bath, brought to vigorous magnetic stirring and 480 μL of a freshly prepared NaBH${}_{4}$ solution (0.1 mol/L) was added, which made the solution to become pale yellow (silver seeds formation) and the reaction was completed within 5 min.

The UV-Vis extinction spectra of the colloidal suspensions (Ag-NSs, Ag-NSPs, and Ag-NPLs) were recorded using a Varian spectrophotometer (Agilent Technologies, Santa Clara, CA, USA), model Cary 50, from 300 to 900 nm. Transmission electron microscopy (TEM) images of Ag-NSs, Ag-NSPs, and Ag-NPLs were taken with a JEOL JEM-1400 transmission electron microscope (JEOL, Peabody, MA, USA) equipped with a Gatan Orius SC1000 camera (JEOL, Peabody, MA, USA). The instrument has a 0.2 nm lattice resolution and magnification range from ×200 to ×1,200,000.

### 3.2. Computer Simulations

The finite difference time domain (FDTD) technique within the commercial software FDTD Solutions (Version 2016, Lumerical Inc., Vancouver, BC, Canada) was employed to perform the optical simulations. The absorption spectra and near-fields were calculated using a total-field scattered-field (TFSF) source. Absorption and scattering were obtained by using two analysis groups, each one of which consists of a box of power monitors: one in the total field region and the other in the scattered field region. Refractive indices for silver nanoparticles $\tilde{n}=n+ik$ in the range considered were obtained by interpolation of the experimental results by Johnson and Christy [40]. Water was considered as the surrounding dielectric medium for all simulations, with refractive index n=1.33. Perfect matched layer (PML) boundary conditions were considered for all end-faces in the simulation process.

## 4. Conclusions

In this letter, we provided a theoretical interpretation for LSPRs in the extinction spectra of Ag-NSPs, Ag-NPLs, and Ag-NSs produced by chemical reduction. Using TEM images for Ag-NSPs, Ag-NPLs, and Ag-NSs, we obtained the averaged geometrical sizes used for numerical simulations with the FDTD technique. The Ag-NSs, for which there is a broad distribution of sizes and shapes, were taken as made by crossed Ag-NRs (see TEM images in Figure 1) for a qualitative understanding of the physics behind the excitation of LSPRs. From the numerical results, we observe that the apparent tail at large wavelengths in the extinction spectra is present for individual Ag-NSs with six-, eight-, and twelve-arms. Hence, this tail for Ag-NSs cannot be attributed solely to aggregation or to the presence of stars with different numbers of arms, unlike the explanations so far given in the literature.
